# Modulation of fast sodium current in airway smooth muscle cells by exchange protein directly activated by cAMP

**DOI:** 10.1152/ajpcell.00417.2023

**Published:** 2023-11-13

**Authors:** Ruth M. Matthews, Eamonn Bradley, Mark A. Hollywood, Fionnuala T. Lundy, Lorcan P. McGarvey, Gerard P. Sergeant, Keith D. Thornbury

**Affiliations:** ^1^Smooth Muscle Research Centre, Dundalk Institute of Technology, Dundalk, Ireland; ^2^School of Medicine, Dentistry and Biomedical Sciences, Wellcome Wolfson Institute for Experimental Medicine, Queen's University Belfast, Belfast, United Kingdom

**Keywords:** airway smooth muscle, cAMP, Epac, Na_v_1.7

## Abstract

Airway smooth muscle (ASM) cells from mouse bronchus express a fast sodium current mediated by Na_V_1.7. We present evidence that this current is regulated by cAMP. ASM cells were isolated by enzymatic dispersal and studied using the whole cell patch clamp technique at room temperature. A fast sodium current, *I*_Na_, was observed on holding cells under voltage clamp at −100 mV and stepping to −20 mV. This current was reduced in a concentration-dependent manner by denopamine (10 and 30 µM), a β-adrenergic agonist. Forskolin (1 µM), an activator of adenylate cyclase, reduced the current by 35%, but 6-MB-cAMP (300 µM), an activator of protein kinase A (PKA), had no effect. In contrast, 8-pCPT-2-O-Me-cAMP-AM (007-AM, 10 µM), an activator of exchange protein directly activated by cAMP (Epac), reduced the current by 48%. The inhibitory effect of 007-AM was still observed in the presence of dantrolene (10 µM), an inhibitor of ryanodine receptors, and when cytosolic [Ca^2+^] was buffered by inclusion of 1,2-bis(*o*-aminophenoxy)ethane-*N*,*N*,*N′*,*N′*-tetraacetic acid, Sigma (BAPTA) (50 µM) in the pipette solution, suggesting that the inhibition of *I*_Na_ was not due to Ca^2+^-release from intracellular stores. When 007-AM was tested on the current-voltage relationship, it reduced the current at potentials from −30 to 0 mV, but had no effect on the steady-state activation curve. However, the steady-state inactivation *V*_1/2_, the voltage causing inactivation of 50% of the current, was shifted in the negative direction from −76.6 mV to −89.7 mV. These findings suggest that cAMP regulates *I*_Na_ in mouse ASM via Epac, but not PKA.

**NEW & NOTEWORTHY** β-adrenergic agonists are commonly used in inhalers to treat asthma and chronic obstructive pulmonary disease. These work by causing bronchodilation and reducing inflammation. The present study provides evidence that these drugs have an additional action, namely, to reduce sodium influx into airway smooth muscle cells via fast voltage-dependent channels. This may have the dual effect of promoting bronchodilation and reducing remodeling of the airways, which has a detrimental effect in these diseases.

## Graphical abstract

**Figure:**
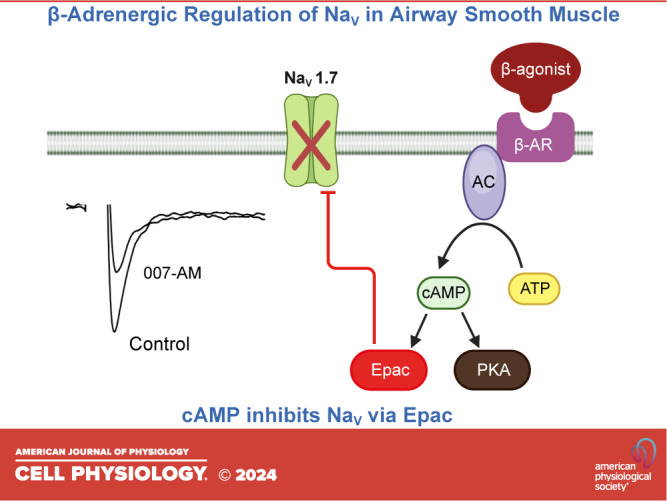

## INTRODUCTION

Fast voltage-gated sodium currents are classified according to their pore-forming α-subunits, of which there are nine known subtypes, designated Na_V_1.1–1.9, encoded by the sodium voltage-gated channels (SCN) gene family (SCN1A–5A and SCN8A–11A) (1). Although normally associated with action potential generating cells such as neurons and cardiac and skeletal muscle myocytes, they have been found in smooth muscle cells in tissues displaying spontaneous myogenic contractions, including lymphatics, gastrointestinal muscle, portal vein, uterus, and vas deferens, where they may play a part in generating electrical activity underling contraction (2–6). Na_V_ currents are also expressed in tonic smooth muscle such as arterial smooth muscle, where they were initially observed in cultured but not freshly dispersed cells, leading to speculation that they were upregulated in cells with an altered phenotype undergoing proliferation or migration during tissue remodeling (7–9). In support of this idea, immunohistochemical staining and quantitative RT-PCR showed significant expression of Na_V_1.7 in aortic tissue following balloon injury, but not in normal aorta. Furthermore, pharmacological inhibition of Na_V_ by TTX, or suppression of Na_V_1.7 expression with small-interfering (si)RNA, inhibited cell migration and secretion of matrix metalloproteinase-2 (MMP-2) (9). However, Na_V_ currents have also been recorded in freshly dispersed cells from pulmonary, mesenteric, and uterine arteries (10–12). In freshly dispersed mesenteric artery cells, expression of Na_V_ currents was critically dependent on the enzymatic dissociation protocol, as the currents were absent when papain was used (11). Although no definite physiological function of Na_V_ channels in normal arterial muscle has been found, several studies have shown that the Na_V_ channel activator, veratridine, can enhance agonist- and KCl-induced contractions and Na_V_ channels may be involved in hypoxia-induced contractions (12–14).

Cultured bronchial smooth muscle cells also express Na_V_ currents, again leading to speculation that they are involved in tissue remodeling (15–17). Indeed, airway smooth muscle cells play an active role in tissue remodeling in the pathogenesis of asthma and chronic obstructive pulmonary disease (COPD), transforming their phenotype into proliferative and secretory cell types (18, 19). Interestingly, dexamethasone, a corticosteroid sometimes used to treat these diseases, suppressed Na_V_1.7 expression in cultured human bronchial myocytes (17). More recently, we have shown that Na_V_1.5 and Na_V_1.7 are also functionally expressed in freshly dispersed rabbit and mouse bronchial myocytes, respectively (20, 21). In these tissues, veratridine was able to enhance agonist contractile responses in vitro, showing that, at least under some circumstances, Na_V_ channels can contribute to bronchial constriction (21).

Along with anticholinergic drugs and corticosteroids, β_2_-adrenergic agonists and phosphodiesterase inhibitors (PDEI) are the mainstay treatments of airway obstruction in asthma and COPD (22, 23). These relieve airway obstruction by increasing intracellular cAMP, which has both bronchodilatory and anti-inflammatory actions (19, 23). Cyclic AMP also regulates Na_V_ currents in neurons, cardiac, and skeletal muscle cells (24), hence, it was of interest in the present study to investigate if it regulates Na_V_ in airway smooth muscle cells. We report that the fast sodium voltage-dependent current, *I*_Na_, in mouse bronchial smooth muscle cells is suppressed by denopamine (a β-adrenergic agonist), forskolin, which stimulates adenylate cyclase and 007-AM (an activator of Epac, exchange protein directly activated by cAMP), but not by 6-MB-cAMP (an activator of PKA, protein kinase A).

## METHODS

### Tissue Dissection and Cell Isolation

All procedures were carried out in accordance with current European Union legislation and with the approval of Dundalk Institute of Technology Animal Use and Care Committee. Male and female C57BL/6 mice (10–16 wk old) were euthanized by intraperitoneal injection of pentobarbitone, and the lungs were removed and placed in oxygenated Krebs solution (*Solution B*). The bronchial tree was exposed by sharp dissection under a microscope to remove surrounding blood vessels and lung tissue. The primary bronchi were removed, cut into small pieces, and placed in Hanks Ca^2+^-free solution (*Solution A*).

Single airway smooth muscle myocytes were isolated using a collagenase/proteinase mixture consisting of (per 5 mL of Hanks Ca^2+^-free solution): collagenase 15 mg (Sigma type 1a), proteinase 1 mg (Sigma type XXIV), BSA 10 mg (Sigma), and trypsin inhibitor 10 mg (Sigma) for ∼5 min at 37°C. They were then placed in Hanks Ca^2+^-free solution (*Solution A*) and stirred for a further 10–20 min to release single relaxed smooth muscle cells. These were plated in Petri dishes containing Hanks Ca^2+^-free solution (with Ca^2+^ added to a concentration of 100 µM) and stored at 4°C for use within 6 h.

### Whole Cell Patch Clamp Recordings

Patch clamp recordings were made using the ruptured patch configuration of the whole cell patch clamp method, as described previously (21). Voltage clamp commands were delivered via an Axopatch 200B patch clamp amplifier (Molecular Devices, Sunnyvale, CA) connected to a Digidata 1322 A AD/DA converter (Axon Instruments) interfaced to a computer running pClamp software (Axon Instruments). When seal resistance exceeded 1 GΩ, access to the cell interior was gained by rupturing the patch, using suction and the “Zap” control on the Axopatch amplifier. Access resistances of 4–6 MΩ were considered acceptable for recording fast voltage-dependent sodium current (*I*_Na_). Traces were leak subtracted in pClamp for analysis and display. Pipette solution used in whole cell experiments was rich in Cs^+^ to block K^+^ currents (*Solution C*). During experiments, the dish containing the cells was superfused with Hanks solution (*Solution D*). In addition, the cell under study was continuously superfused (*Solution D*) by means of a close delivery system consisting of a pipette (tip diameter 200 µm) placed ∼300 µm away. This could be switched, with a dead-space time of <5 s, to a solution containing a drug. All patch clamp experiments were carried out at room temperature.

**Figure 1.**
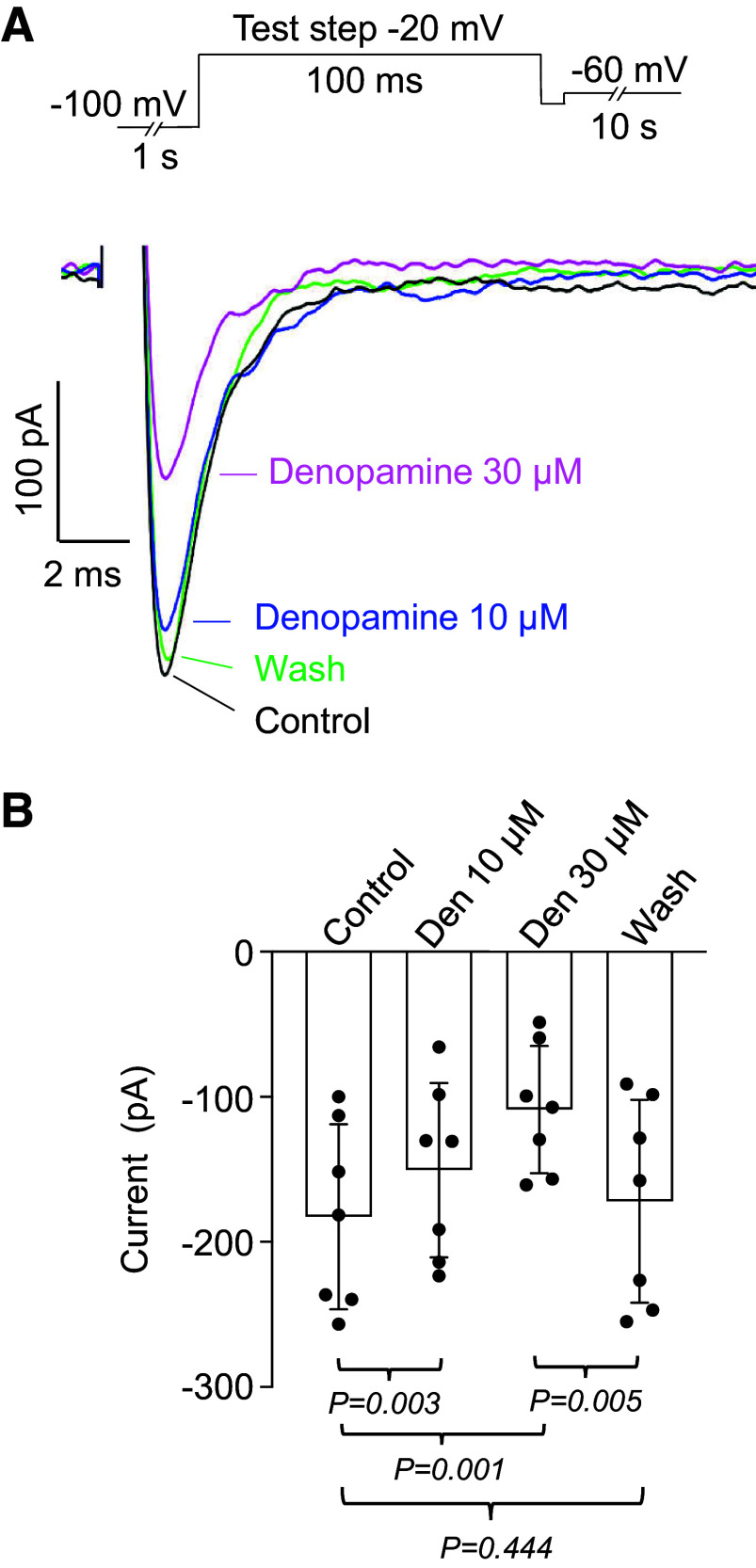
Effect of denopamine on *I*_Na_. *A*: inset shows voltage protocol (not to scale). Current during control is shown by the black line and washout (Wash) by the green line. Denopamine (10 µM, blue line) and (30 µM magenta line) reduced the current. *B*: summary of seven similar experiments, *n* = 7 cells from *N* = 7 animals (6 M, 1 F). *P* values were obtained using ANOVA, followed by Tukey’s test for multiple comparisons. F, female; M, male.

### Drugs and Solutions

The following solutions were used. Concentrations in mM are given in parentheses.

*Solution A* (Ca^2+^ free Hanks solution for cell dispersal), mM: NaCl (125), KCl (5.4), glucose (10), sucrose (2.9), NaHCO_3_ (4.2), KH_2_PO_4_ (0.4), NaH_2_PO_4_ (0.3), HEPES (10), and pH was adjusted to 7.4 using NaOH.

*Solution B* (Krebs solution), mM: NaCl (120), KCl (5.9), NaHCO_3_ (25), NaH_2_PO_4_.2H_2_O (1.2), glucose (5.5), MgCl_2_ (1.2), CaCl_2_ (2.5), and pH was adjusted to 7.4 by bubbling the solution with 95% O_2_ – 5% CO_2_ continuously.

*Solution C* (pipette solution for whole cell patch clamp recordings), mM: CsCl (133), MgCl_2_.6H_2_O (1.0), EGTA (2.0), HEPES (10.0), Na_2_ATP (1.0), NaGTP (0.1), Na_2_ phosphocreatine (2.5), and pH adjusted to 7.2 using CsOH.

*Solution D* (Hanks, bath solution for patch clamp recording), mM: NaCl (125), KCl (5.36), glucose (10), sucrose (2.9), NaHCO_3_ (4.17), KH_2_PO_4_ (0.44), NaH_2_PO_4_ (0.33), MgCl_2_.6H_2_O (0.5), CaCl_2_.2H_2_O (1.8), MgSO_4_.7H_2_O (0.4), HEPES (10), and pH adjusted to 7.4 with NaOH.

The following drugs were used: 6-MB-cAMP (BioLog), 8-pCPT-2-O-Me-cAMP-AM (007-AM, Biolog), forskolin (ABCAM), BAPTA (1,2-bis(*o*-aminophenoxy)ethane-*N*,*N*,*N′*,*N′*-tetraacetic acid, Sigma), denopamine (Santa Cruz), and dantrolene (Sigma).

### Data Analysis and Statistics

All of the experiments in each experimental series were taken from different animals. Only one cell per animal was used in each experimental series. Hence, “*n*” refers to the number of cells and is equivalent to the number of animals used. In each case, the sex is noted as F, female or M, male. Data were analyzed using Prism software (Graphpad). Summary data are presented as means ± SD, and individual data points are displayed. As no differences were noted between cells isolated from males and females, data from both sexes were pooled.

Statistical comparisons were made using either Student’s paired *t* test or, if three experimental groups were compared, repeated measures one-way ANOVA followed by the Tukey’s post hoc test for multiple comparisons, taking *P* < 0.05 as significant. *P* values displayed on graphs are rounded to three decimal places. For data requiring statistical comparisons a minimum sample size of *n* = 6 was chosen, based on previous power calculations and extensive experience. Current-voltage (*I-V*) relationships were compared using two-way ANOVA, followed by Bonferroni’s test for multiple comparisons.

All experiments used an in vitro within subject (paired samples) experimental model, comparing drug effects with baseline or determining incremental concentration-effect relationships. Randomization was not considered necessary in such experiments. Blinding of the operator was not possible because of the knowledge required to run the experimental protocols and because responses observed by the operator to manage the experiment permitted inferences about the treatment. Electrophysiological data were analyzed by the operator using pCLAMP software (Molecular Devices) to electronically measure either peak responses found independently by the software or at predetermined time points.

Sigmoidal activation curves were fitted with a Boltzmann equation: *g/g*_max_ = 1/{1 + exp[±(*V*_1/2_ – *V*_m_)/*K*]}, where *V*_1/2_ is membrane potential at which there was half-maximal activation, *K* is the slope factor, *V*_m_ is the test potential, *g* is conductance, and *g*_max_ is maximal conductance. Conductance (*g*) was calculated as follows: *g* = *I*/(*V*_m_ − *E*_Na_), where *E*_Na_ is the calculated Nernst potential for Na^+^ and *I* is the current recorded. Inactivation curves were fitted with a similar Boltzmann function: *I/I*_max_ = 1/{1 + exp[±(*V*_1/2_ − *V*_c_)/*K*]}, where *I* is the current recorded at the test step, *I*_max_ is the maximal current recorded, *V*_c_ is the conditioning potential (see results), and *K* is the slope factor. The *V*_1/2_ of activation and inactivation for control and drug-treated cells were compared using the Extra sum-of-squares *F* test.

## RESULTS

Fast voltage-dependent sodium current (*I*_Na_) in mouse bronchial myocytes is mainly carried by Na_V_1.7 (21). The protocol to study the sensitivity of this current to drugs was similar to that used previously (21, and *inset* Fig. 1*A*) and involved holding cells at −60 mV, then stepping to −100 mV for 1 s to remove inactivation, followed by a depolarizing step to −20 mV to evoke maximal current. This was repeated at 10 s intervals as a drug was washed in until it had its maximal effect. Although in human ASM β_2_-adrenoceptors are predominantly expressed over β_1_-adrenoceptors, in mice ASM β_1_-adrenoceptors predominate (25). In the example in Fig. 1*A*, the effect of denopamine, a β_1_-adrenoceptor agonist, was examined. When 10 µM denopamine was washed in, a reduction in current amplitude of ∼13% was observed, whereas 30 µM denopamine caused a reduction of ∼52%. When denopamine was washed out, the current recovered to the control level. Summary data from seven experiments confirmed these observations, with both 10 and 30 µM denopamine causing a significant, concentration-dependent, reduction in current.

**Figure 2.**
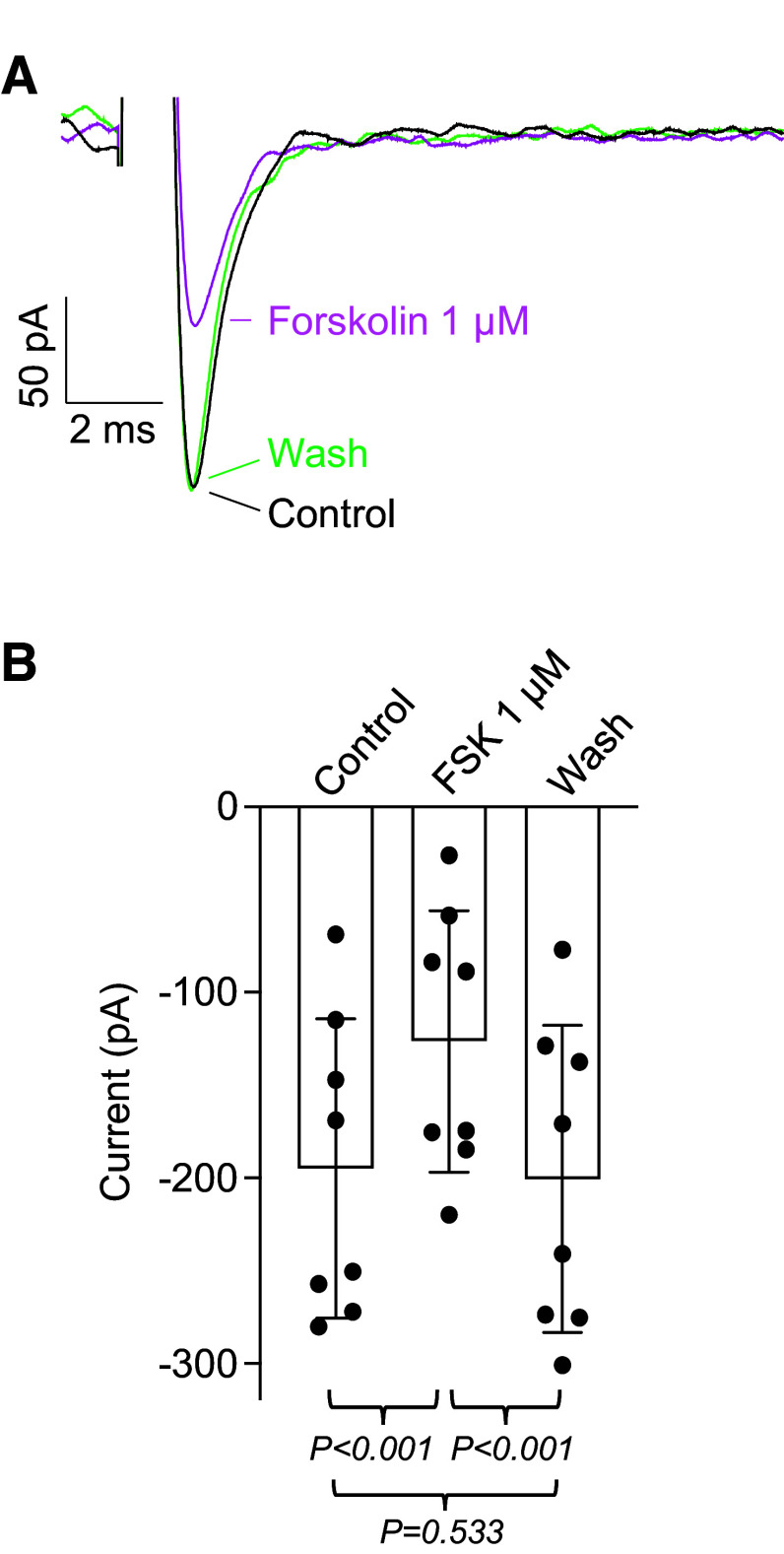
Effect of forskolin on *I*_Na_. *A*: voltage protocol as in Fig. 1. Currents during control and washout (Wash) are shown as black and green lines, respectively. Forskolin 1 µM (magenta line) reduced the current by 48%. *B*: summary of eight similar experiments, *n* = 8 cells from *N* = 8 animals (7 M, 1 F). *P* values were obtained using ANOVA, followed by Tukey’s test for multiple comparisons. F, female; FSK, forskolin; M, male.

Since the effects of β-adrenoceptor stimulation in airways smooth muscle are mainly due to the activation of adenylate cyclase (25), we examined the effect of forskolin (1 µM) a direct activator of adenylate cyclase. Figure 2*A* shows an example where forskolin was applied and then washed out. In this example, forskolin reduced the current by ∼48% and then it fully recovered following washout of the drug. In the summary data from eight experiments shown in Fig. 2*B*, there was an overall reduction of ∼35% in the mean current amplitude. These results suggest that cAMP has a role in regulating Na_V_ in these cells.

**Figure 3.**
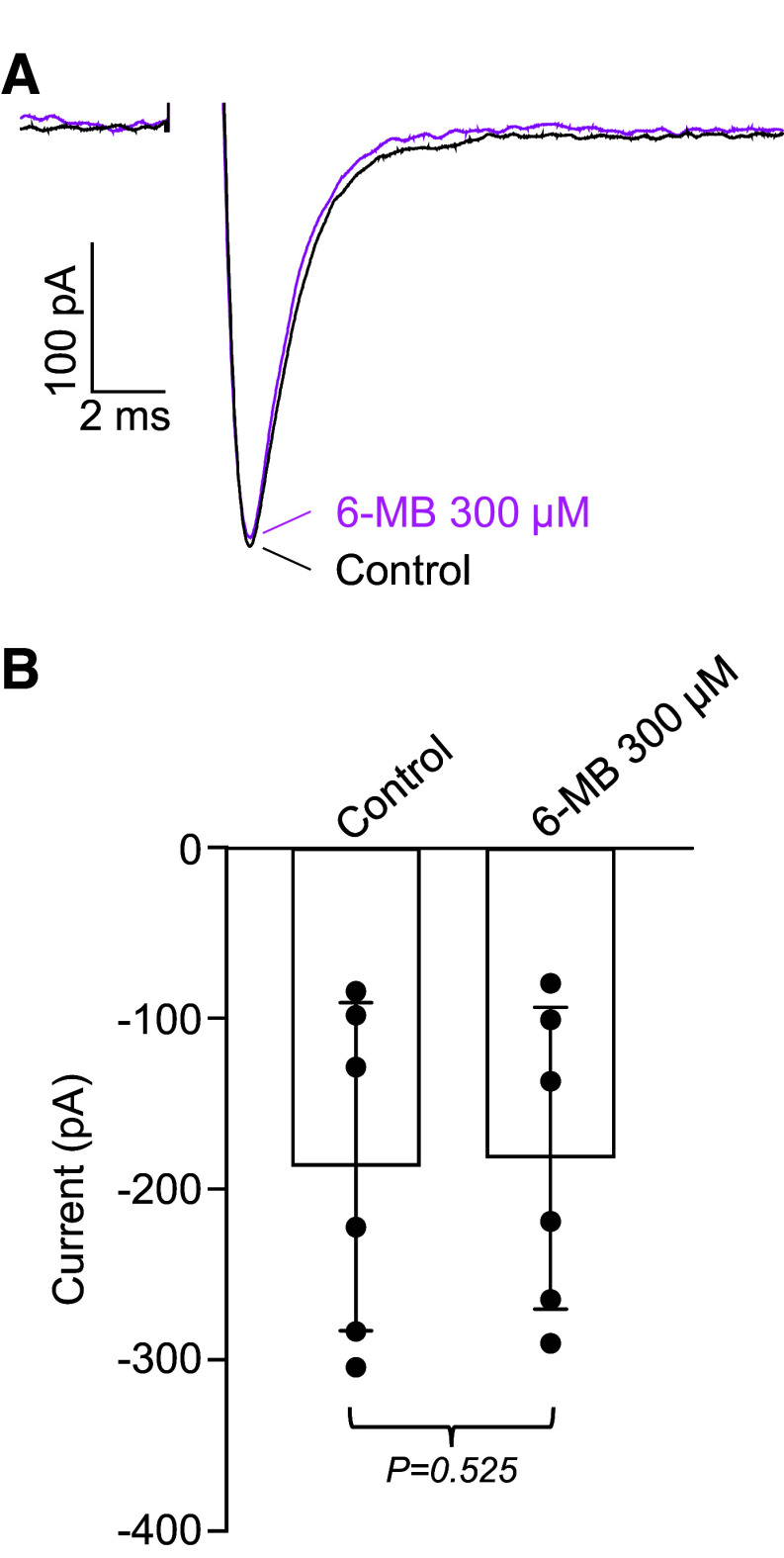
Effect of 6-MB-cAMP (6MB) on *I*_Na_. *A*: voltage protocol as in Fig. 1. Current during control is shown as a black line. 6-MB-cAMP (300 µM, magenta line) had no effect on current amplitude. *B*: summary of six similar experiments, *n* = 6 cells from *N* = 6 animals (6 M). *P* value was obtained using Student’s paired *t* test (two tailed). M, male.

The main downstream effectors for cAMP are the classical protein kinase A (PKA) pathway (26) and the more recently discovered exchange protein directly activated by cAMP (Epac; 27, 28). Hence, we investigated the role of these cAMP-sensing proteins using highly selective activators, 6-MB-cAMP for PKA and 007-AM for Epac (29). Figure 3*A* shows an example where 6-MB-cAMP (300 µM) was applied, and had a minimal effect, if any. Summary data in six similar experiments confirmed the lack of effect of 6-MB-cAMP (Fig. 3*B*). As we have previously shown that 6MB (300 μM) inhibited carbachol-induced Ca^2+^ oscillations in mouse airway smooth muscle cells and, at 100 μM, reduced purinergic receptor-mediated contractions of mouse detrusor by >60%, we conclude from the present experiments that PKA did not modulate *I*_Na_ (30, 31).

**Figure 4.**
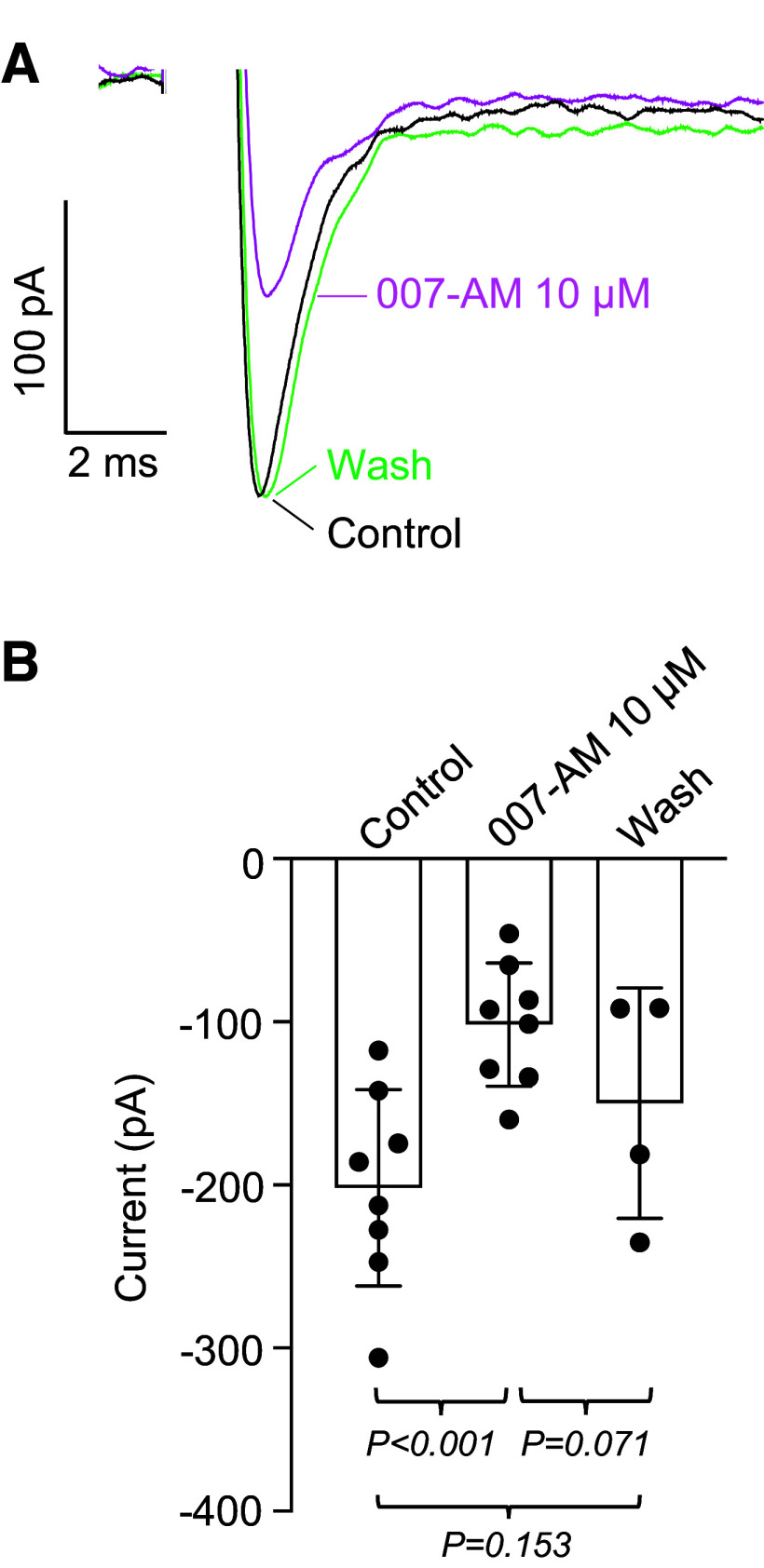
Effect of 007-AM on *I*_Na_. *A*: voltage protocol as in Fig. 1. Currents during control and washout (Wash) are shown as black and green lines, respectively. 007-AM (10 µM, magenta line) reduced the current by 48%. *B*: summary of eight similar experiments, *n* = 8 cells from *N* = 8 animals (4 M, 4 F). Note: in four of the cells, the seal was lost before washout was complete. *P* values were obtained using ANOVA, followed by Tukey’s test for multiple comparisons. F, female; M, male.

**Figure 5.**
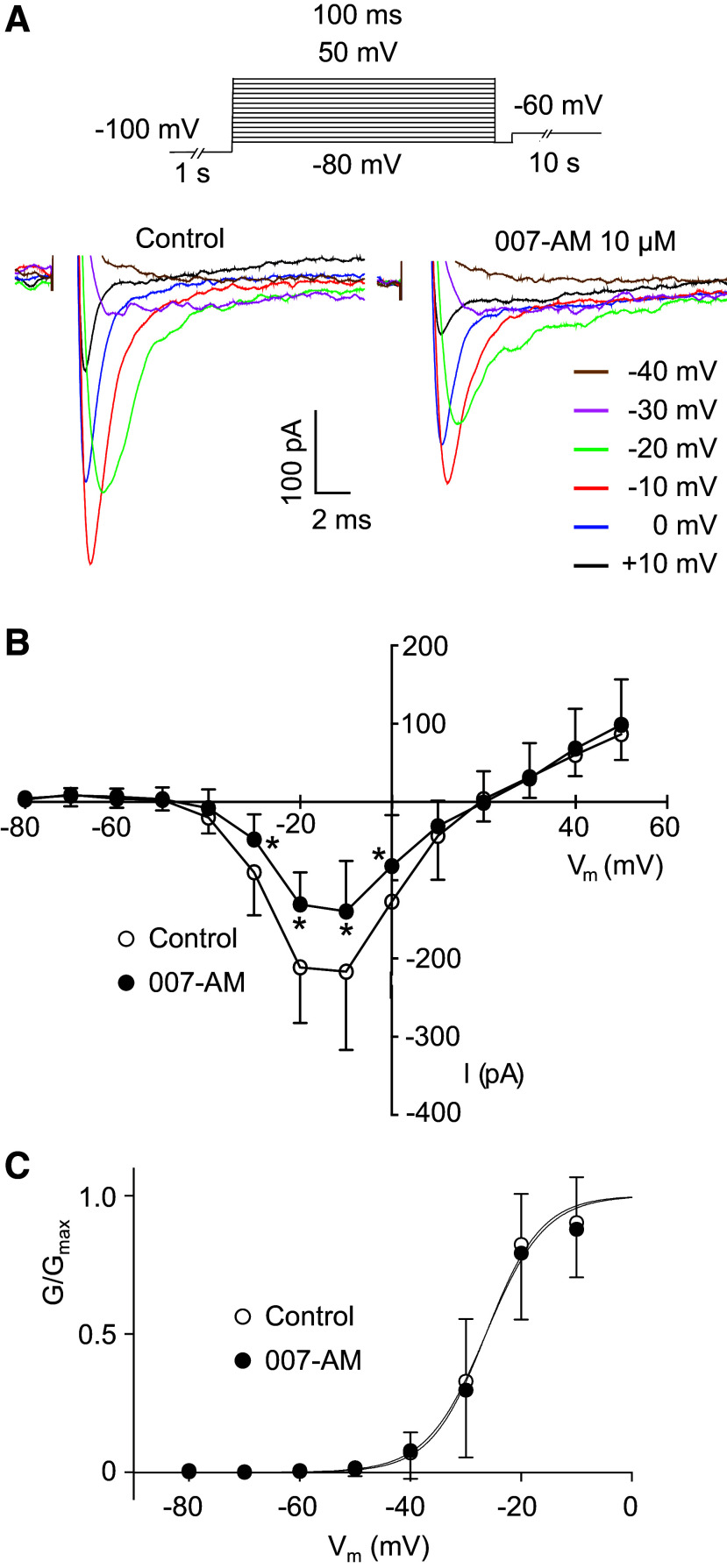
Effect of 007-AM on *I*_Na_ current-voltage (*I-V*) relationship and activation curve. *A*: *I-V* relationship obtained in a cell using the voltage protocol shown in *inset*. 007-AM reduced the current amplitude uniformly at all potentials. *B*: summary of seven similar experiments, *n* = 7 cells from *N* = 7 animals (4 M, 3 F). *P < 0.001, obtained using two-way ANOVA followed by Bonferroni’s test for multiple comparisons. *C*: activation curves derived from the data in *B*. Control *V*_1/2_ of activation = −26.7 mV (95% Cl, −28.3 to 25.2); 007-AM *V*_1/2_ of activation = −25.9 mV (95% Cl, −27.7 to −24.0); these values were not significantly different, *P* = 0.482, extra sum of squares *F* test. F, female; M, male.

**Figure 6.**
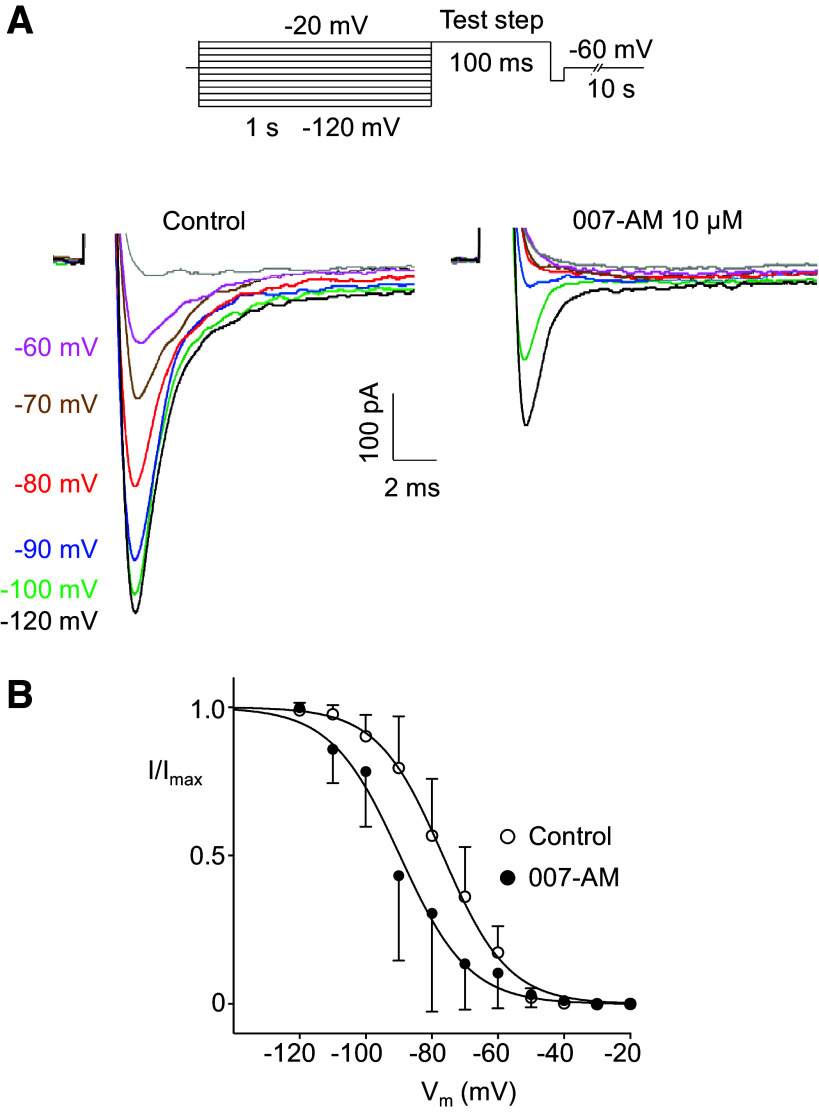
Effect of 007-AM voltage-dependent inactivation of on *I*_Na_. *A*: voltage-dependent inactivation of a cell. The protocol (*inset*) involved subjecting the cell to a series of conditioning steps before stepping to a test potential of −20 mV in each case. The voltages indicated by the colored text refer to the conditioning step that preceded each trace. 007-AM reduced the current amplitude but also caused inactivation to occur over a more negative range of potentials, e.g., compare inactivation in response to the −90 mV conditioning step (blue traces) in each case. *B*: summary plot of mean inactivation curves obtained from six experiments, *n* = 6 cells from *N* = 6 animals (2 M, 4 F). The *V*_1/2_ of inactivation in control was −76.6 mV (95% Cl, −78.5 to 74.8 mV) and in 007-AM was −89.7 mV (95% Cl, −92.8 to 86.5 mV); these values were significantly different, *P* < 0.0001, extra sum of squares *F* test. F, female; M, male.

In contrast, 007-AM (10 µM) reduced the current amplitude in a similar manner to denopamine and forskolin. In the example shown in Fig. 4*A*, the reduction was ∼48% and in a series of eight experiments, the mean reduction was 50% (Fig. 4*B*). The effect of 007-AM was further examined on the current-voltage relationship (*I-V*) and on steady-state voltage-dependent activation and inactivation. Figure 5*A* shows an *I-V* relationship generated using the protocol shown in the *inset*. The protocol consisted of stepping to conditioning potentials of −100 mV for 1 s to remove inactivation, followed by a series of test steps ranging from −80 mV to + 50 mV for 100 ms at 10 s intervals. Figure 5*A* shows a series of currents in response to these steps before and after 007-AM (10 µM). 007-AM reduced the currents at all potentials, confirmed by the summary data for seven experiments shown in Fig. 5*B*, where the reduction across the voltage range appeared to be uniform. These reductions were statistically significant for the values recorded at voltages −40 mV through to 0 mV. Figure 5*C* shows activation curves derived by correcting the data in Fig. 5*B* for driving force. The *V*_1/2_ of activation for control was −26.7 mV and for 007-AM, it was −25.9 mV. These values were not significantly different, confirming that 007-AM had no effect on activation. In contrast, 007-AM shifted the steady-state voltage dependence of inactivation. This was examined by exposing cells to conditioning potentials of −120 mV to −20 mV for 1 s, before stepping to a test potential of −20 mV for 100 ms (*inset* Fig. 6*A*). Figure 6*A* shows recordings from a cell exposed to this protocol before and after 007-AM (10 µM). Notably, the currents were of smaller amplitude in 007-AM, but also the current inactivated over a more negative range of potentials. For example, compare the current following the −90 mV conditioning potential in each case (blue lines). Before exposure to 007-AM, the current was 85% of the maximal current recorded in control, whereas in 007-AM it was only 13% of maximal recorded in the drug. In the summary data in Fig. 6*B*, there was a shift in the negative direction of the voltage axis in the presence of 007-AM. Comparison of the *V*_1/2_ of inactivation in control and in the drug showed a significant negative shift from −76.6 mV to −89.7 mV.

**Figure 7.**
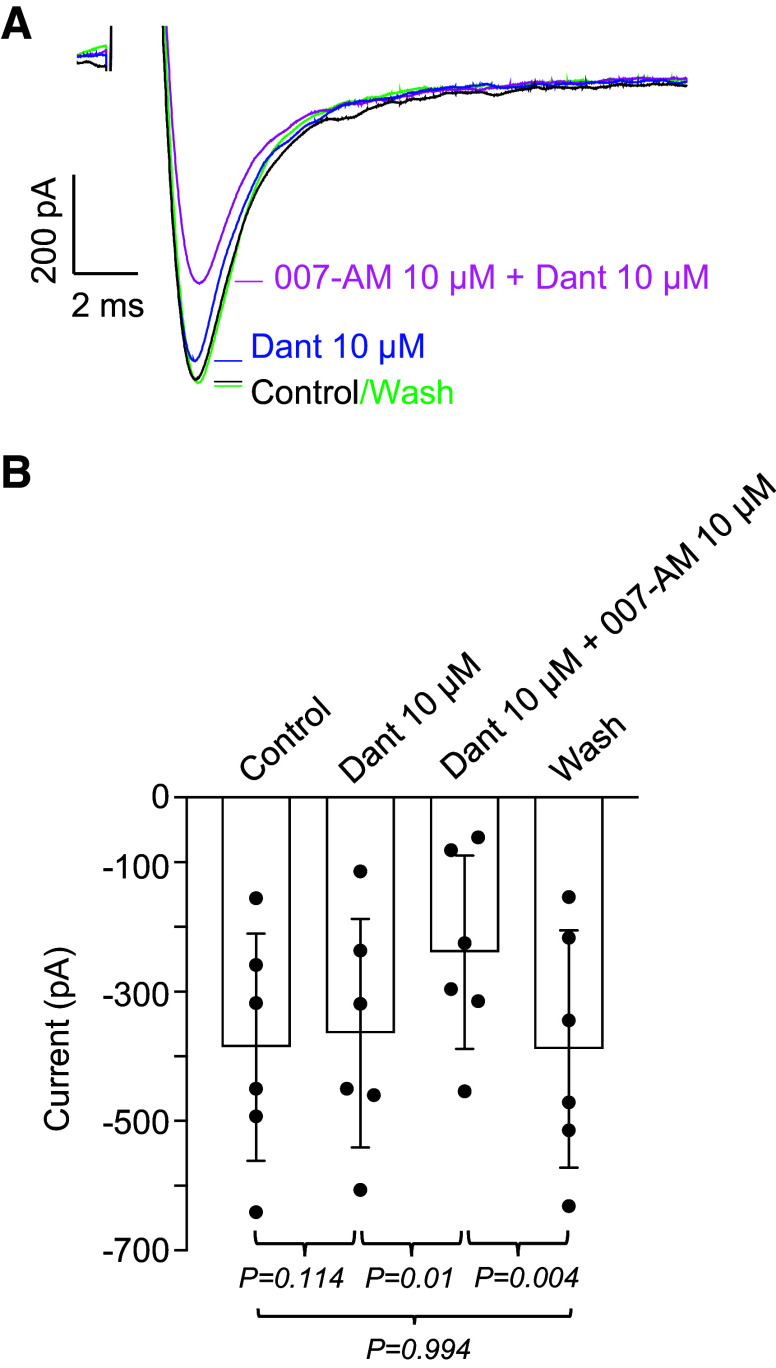
Lack of effect of dantrolene on the ability of 007-AM to reduce *I*_Na_. *A*: voltage protocol was as in Fig. 1. Currents during control and washout (Wash) are shown as black and green lines, respectively. Dantrolene (Dant, 10 µM, blue line) had a minimal effect on the current, whereas 007-AM (10 µM) in the presence of dantrolene (magenta line) reduced the current. *B*: summary of six similar experiments, *n* = 6 cells from *N* = 6 animals (3 M, 3 F). *P* value was obtained using ANOVA, followed by Tukey’s test for multiple comparisons. F, female; M, male.

In cardiac (32) and skeletal muscle (33) activation of Epac also caused a reduction in the amplitude of *I*_Na_. In these studies, the effect was blocked by dantrolene, hence the mechanism was believed to be dependent on Ca^2+^ release from ryanodine receptors (RyR) that, in turn, caused Ca^2+^-mediated inhibition of the *I*_Na_. Since Epac can also cause Ca^2+^-release via RyR in smooth muscle (34), we decided to investigate if Ca^2+^ release via RyR could account for the inhibition of *I*_Na_ by 007-AM. In the example in Fig. 7*A*, the effect of dantrolene (10 µM) was examined both before and during addition of 007-AM. Dantrolene itself appeared to cause a small reduction in *I*_Na_, but notably, 007-AM was still able to exert its effect in the presence of dantrolene, followed by full recovery of *I*_Na_ amplitude on washout. A summary of six similar experiments is presented in Fig. 7*B.* In these data there was no significant effect of dantrolene on *I*_Na_, but 007-AM was still able to significantly reduce *I*_Na_ by 35% (compared with dantrolene alone), with full recovery evident on washout.

**Figure 8.**
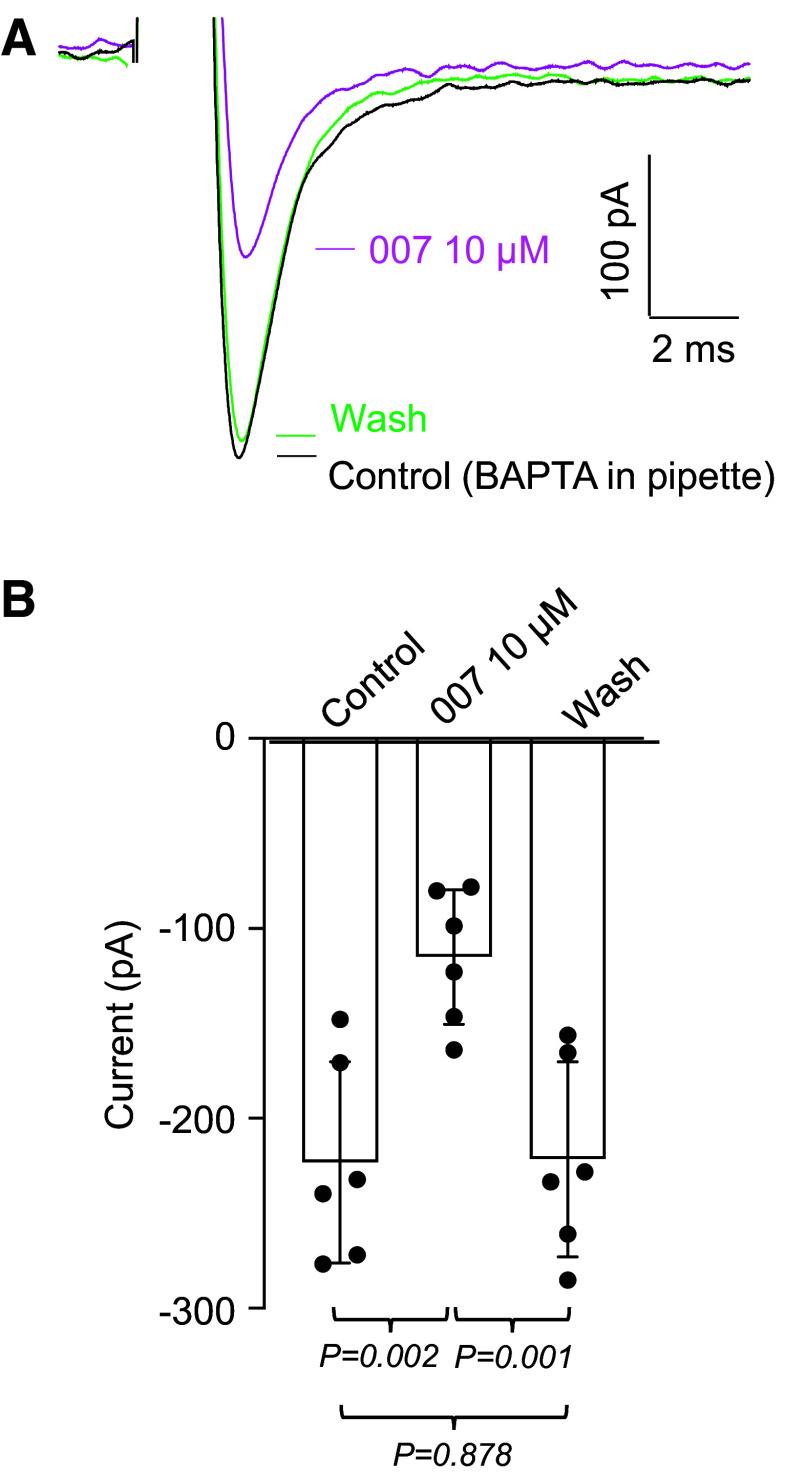
Effect of 007-AM (10 µM) on *I*_Na_ when BAPTA (50 µM) was included in the pipette solution. *A*: voltage protocol as in Fig. 1. Currents during control and washout (Wash) are shown as black and green lines, respectively. 007-AM (10 µM, magenta line) reduced the current by 50%. *B*: summary of six similar experiments, *n* = 6 cells from *N* = 6 animals (6 F). *P* value was obtained using ANOVA, followed by Tukey’s test for multiple comparisons. BAPTA, 1,2-bis(*o*-aminophenoxy)ethane-*N*,*N*,*N′*,*N′*-tetraacetic acid; F, female.

We further tested if the reduction in *I*_Na_ by 007-AM could have been due to a change in intracellular Ca^2+^ concentration by including BAPTA (50 µM), a fast Ca^2+^ buffer, in the pipette solution. Although we had already included EGTA (2 mM) in the pipette solution, there was a concern that this relatively slow Ca^2+^ buffer might not have been fast enough to capture Ca^2+^ ions if they were released close to their binding site on the Na_v_ channels (33). Figure 8*A* shows an example of an experiment where BAPTA was included in the pipette solution. Under these conditions, 007-AM was still able to exert its full effect on *I*_Na_. Figure 8*B* shows a summary of six experiments, where 007-AM was still able to reversibly reduce the amplitude of *I*_Na_ by 48%.

## DISCUSSION

Cyclic AMP is an important inhibitory second messenger in airways, which causes smooth muscle relaxation as well as anti-inflammatory effects, including inhibition of smooth muscle proliferation and remodeling, characteristic of chronic airway diseases such as asthma and COPD (35). Consequently, boosting intracellular cAMP concentration with β_2_-adrenergic agonists and/or PDEIs has, for several decades, been one of the major therapeutic strategies for treating these diseases (22, 23). Many of the intracellular effects of cAMP in airways smooth muscle are mediated by PKA, which phosphorylates multiple proteins that promote relaxation by either reducing intracellular Ca^2+^ concentration or by reduced Ca^2+^-sensitivity of the contractile proteins (24). However, PKA-independent effects of β_2_-adrenergic agonists on relaxation and proliferation have also been reported and these have now been convincingly attributed to activation of Epac (27, 36). Roscioni et al. (27) showed that Epac-specific activators were effective at inhibiting cholinergic-mediated contractions, whereas a PKA-specific inhibitor only weakly inhibited isoproterenol-induced relaxations. This was achieved, at least in part, by altering Ca^2+^-sensitivity by shifting the balance of phosphorylation from RhoA to Rac1, resulting in a reduction in phosphorylation of myosin light chain (MLC). However, a recent study from our laboratory showed that Epac could also reduce cytosolic Ca^2+^ in isolated mouse ASM myocytes (30). In this study forskolin, PGE2, and the EP_2_R-specific agonist (R)-butaprost were all able to abolish CCh-induced Ca^2+^ oscillations, but these effects were only partially blocked with the PKA-specific antagonist, RP-8-CPT-cAMPs. Similarly, 6-MB-cAMP alone was not able to fully mimic the effect of forskolin, however, when applied together with 007-AM both agents were able to abolish the CCh-mediated Ca^2+^ oscillations. These results suggested that Epac not only reduces the Ca^2+^-sensitivity in ASM but also modulates the underlying Ca^2+^ signal itself.

Na_V_ channels are also regulated by phosphorylation by various kinases, including PKA but the effects are complex, depending on the Na_V_ channel subtype, its splice variant, and the expression system in which the channels are studied (24). For example, in cardiac myocytes, where Na_V_1.5 is the predominant subtype, activation of β-adrenergic receptors had variable effects, though most studies reported an increase in current, due in part to a negative shift in activation kinetics, but also a slower onset response believed to be due to increased trafficking of Na_V_ to the plasma membrane (24). In contrast, in a variety of brain neurons that express Na_V_1.1 and Na_V_1.2, PKA-activation reduces *I*_Na_ without changing its steady-state properties (24, 37). The main Na_V_ α-subtype expressed in mouse and human ASM is Na_V_1.7 (16, 17, 21). In neurons, Na_V_1.7 has long and short splice variants. PKA activation reduced the amplitude of currents encoded by the long splice variant when expressed in *Xenopus* oocytes (24, 38), but had no effect when it was expressed in mammalian cells (24, 39). However, in mammalian cells, PKA shifted the activation *V*_1/2_ of the short splice variant to more negative potentials (39).

In contrast to PKA, there are few published studies where Epac-mediated regulation of Na_V_ channels have been investigated. In the heart, β-AR stimulation with isoproterenol results in calcium/calmodulin-dependent protein kinase II (CaMKII)-mediated and non-CaMKII-mediated regulation. CaMKII can directly phosphorylate the Na_V_ channels, resulting in enhancement of the late (noninactivating) component of *I*_Na_ and a negative shift in steady-state inactivation of −9 mV (40). A selective Epac-activator resulted in autophosphorylation of CaMKII and enhancement of the late current comparable with isoproterenol, suggesting that Epac was involved. These effects were blocked by paclitaxel, a chemotherapeutic agent that causes tubulin polymerization, suggesting that microtubules are required for Epac-mediated activation of CaMKII (40). Two other studies describe a reduction in amplitude of *I*_Na,_ with no change in *V*_1/2_ values for activation and inactivation following Epac-activation in the cardiac (Na_V_1.5) and skeletal muscle myocytes (Na_V_1.4), respectively (32, 33). In both cases, the effects were completely reversed by dantrolene, suggesting that Epac-mediated release of Ca^2+^ from RyR2 and RyR1, respectively, resulted in Ca^2+^-dependent inactivation of *I*_Na_.

In the present study, activation of β-AR with denopamine, adenylate cyclase with forskolin, and Epac with 007-AM all resulted in an ∼50% reduction in the maximum *I*_Na_ amplitude in isolated mouse bronchial myocytes. In contrast, the specific PKA activator, 6-MB-cAMP, had no effect, suggesting that cAMP-mediated modulation of *I*_Na_ in these cells is entirely via Epac. In our experiments, the Epac-mediated reduction in current amplitude was not affected by either dantrolene or BAPTA, a fast Ca^2+^ buffer that would be expected to be able to capture Ca^2+^ ions even if they were released from RyRs in close proximity to the Na_V_ channels (41). Moreover, in a previous study, we did not observe 007-AM to induce Ca^2+^ sparks in isolated mouse bronchial myocytes, suggesting that the reduction in *I*_Na_ amplitude in the present study was not due to release of Ca^2+^ (30).

The effects of 007-AM on steady-state activation and inactivation kinetics were studied further, showing that the *V*_1/2_ for activation was unaffected, whereas the *V*_1/2_ for inactivation was shifted in the negative direction by −15 mV. The negative shift in inactivation may partly account for the reduction in *I*_Na_ amplitude, but is not likely to account for the whole effect, as in the inactivation protocols the current, even following the most negative conditioning potential of −120 mV, was still reduced by ∼50% (Fig. 6). However, some caution is required with this conclusion because it was not possible to assess the effects of prepotentials more negative than −120 mV, as the cells did not withstand these voltages.

Although we do not know how Epac acts on Na_V_1.7 channels to inhibit their activity, several studies suggest that Akt/protein kinase B (PKB) might be involved. First, Epac causes phosphorylation and activation of Akt/protein kinase B (PKB), whereas PKA inactivates it (42). Second, inhibition of Akt/PKB resulted in enhancement of Na_V_1.6-mediated currents (43), whereas Akt/PKB directly phosphorylated Na_V_1.1 channels and inhibited their associated currents (44). The way that Akt/PKB regulates these channels may not be the same, as in the latter case the voltage-dependence of inactivation and activation were shifted (to the left and right, respectively), but the voltage-dependent kinetics of the Na_V_1.6-mediated currents were not affected. Nevertheless, these studies suggest that a potential role for Akt/PKB in regulating *I*_Na_ in airway smooth muscle warrants further study.

In the present study, we have provided evidence that cAMP, acting via Epac, can inhibit Na_V_ current in mouse bronchial smooth muscle cells. Although substantial *I*_Na_ currents can be recorded in these cells, their role has not been clearly established. However, there has been much speculation that Na_V_ is involved in the transformation of smooth muscle cells to a secretory and proliferative phenotype, important in airway remodeling in disease states such as asthma and COPD (15–17). Indeed, there is now overwhelming evidence that Na_V_ currents are important for cell migration and invasiveness in a wide range of cancer cells (45–48). Also, it is interesting that Epac has an antiproliferative effects on airway smooth muscle cells (36), and Epac is downregulated by cigarette smoke extract and in patients with COPD (49), suggesting that a loss of the suppressive effects of Epac contributes to remodeling. As we have shown that Epac suppresses *I*_Na_ in bronchial myocytes, we speculate that loss of Epac-mediated regulation of Na_V_ channels may contribute to tissue remodeling in airways diseases. Although the inactivation *V*_1/2_ of *I*_Na_ might suggest that little current is available under physiological conditions, it is possible that its properties change during inflammation, or that the membrane potential may shift under these circumstances. For example, we were able to demonstrate contractile effects of veratridine, a Na_V_ channel activator, in the presence of prostaglandin E2, which is likely to cause membrane hyperpolarization (21). It is possible, therefore, that under some circumstances, suppression of Na_V_ contributes to the relaxant effects of β-adrenergic agonists in airways smooth muscle.

## DATA AVAILABILITY

Data will be made available upon reasonable request.

## GRANTS

This work was performed as part of the BREATH project, funded by the European Commission Interreg VA Health and Life Science Program (Grant No.: INT-VA/045).

## DISCLOSURES

No conflicts of interest, financial or otherwise, are declared by the authors.

## AUTHOR CONTRIBUTIONS

R.M.M., E.B., M.A.H., G.P.S., and K.D.T. conceived and designed research; R.M.M. and E.B. performed experiments; R.M.M. and K.D.T. analyzed data; R.M.M., M.A.H., F.T.L., L.P.M., G.P.S., and K.D.T. interpreted results of experiments; R.M.M. and K.D.T. prepared figures; K.D.T. drafted manuscript; R.M.M., M.A.H., F.T.L., L.P.M., G.P.S., and K.D.T. edited and revised manuscript; R.M.M., E.B., M.A.H., F.T.L., L.P.M., G.P.S., and K.D.T. approved final version of manuscript.
